# Construction and hydrophilic modification of dual-network structured nonwoven/UHMWPE composite membranes for water processing

**DOI:** 10.1039/d3ra00920c

**Published:** 2023-06-20

**Authors:** Yunan Zhu, Rong Liu, Xiaoxiao Xu, Yijun Fu, Jiamu Dai, Wei Zhang, Guangyu Zhang, Junxiong Zhang, Xiaomin Ma, Pin Chen

**Affiliations:** a School of Textile & Clothing, National & Local Joint Engineering Research Center of Technical Fiber Composites for Safety and Health, Nantong University Nantong 226019 P. R. China rong063@ntu.edu.cn +86 21 85012837; b National Equipment New Material & Technology (Jiangsu) Co., Ltd Suzhou 215100 P. R. China; c Jiangsu Jinni Engineering Fabric Co., Ltd Nantong 226019 P. R. China

## Abstract

Water pollution caused by the continuous development of industrialization has always been a common concern of mankind. Herein, a novel strategy to fabricate a high-performance composite membrane based on dual-network structured nonwoven net/UHMWPE nanopores *via* a thermal phase separation and composite technique is reported. By thermal phase separation of ultra-high-molecular weight polyethylene (UHMWPE)/liquid paraffin (LP), this approach enables 3D nanopores to tightly bond with a nonwoven net to form a dual-network structure. The dual-network composite membrane possesses the integrated features of pore structure and high porosity (89.9%). After modification with hyperbranched polymers (HBPs), the composite membrane with the desirable surface chemistry achieves high-efficiency filtration (water flux = 1054 L m^−2^ h^−1^, rejection rate = 50 nm PS nanospheres almost close to 100%, and antibacterial properties). The fabrication of such composites may provide new insights into the design and development of high-performance filtration and separation materials for various applications.

## Abstract 2

Water pollution caused by the continuous development of industrialization has always been a common concern of mankind.

## Graphical abstract

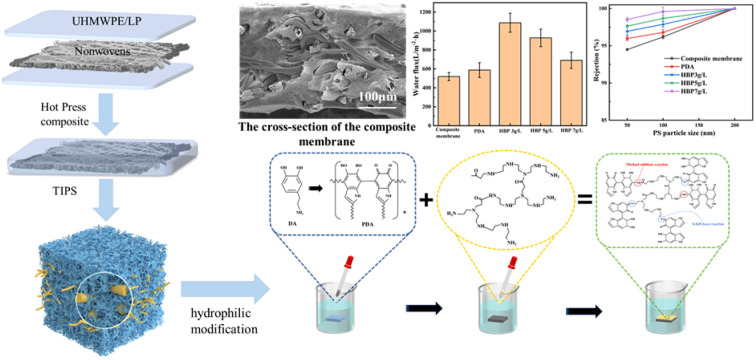

## 1. Introduction

With the shortage of global water resources which is increasingly serious, wastewater treatment has become the main way to recycle and regenerate water resources.^1–4^ Compared with the traditional separation technology, membrane separation technology has been widely used in the field of wastewater treatment because of its high efficiency, low energy consumption and wide range of applications.^5–8^ Moreover, nonwoven materials have developed rapidly due to their short process, fast production speed, high output and low cost.^9–11^ Nonwoven filters have become a hot topic in the field of filtration. However, the pores of traditional industrial nonwoven filters are distributed between 10 and 160 μm, and the minimum particle size that can be intercepted is 0.5–10 μm; the application of nonwoven filters in the field of water filtration is limited.^12–19^

Nowadays, dual-network structures inspired by spider webs attract the attention of many researchers, and numerous studies show that the continuous ultrafine fibrous structure can yield substantial improvements in material utilization and resulting properties.^20^ Liu *et al.*^21^ prepared a continuously welded 2D nanonetwork with a fiber diameter of 20 nm, and topological Steiner tree structures were assembled into membranes with high-efficiency and transparent nanonetwork air filters. This spider web-like membrane showed excellent filtration efficiency, which could filtrate PM2.5 with a removal efficiency of >99.6% and a pressure drop of <5 Pa. Zhang *et al.*^22^ adopted ejection and deformation/phase separation and the charged droplets from a Taylor cone were tailored to make them evolve and assemble into 2D NF-nets inspired by spider webs. The resulting SWING filters can remove MPPS PM0.3 with high efficiency of >99.9%, an atmospheric pressure <0.1%, and high transparency of >82.0%. Therefore, constructing spider web-like dual-network structures on nonwoven materials by thermal phase separation (TIPS) is a strategy to improve the filtration accuracy of nonwoven materials.

TIPS is a process in which the polymer is melted and blended with the diluent to form a homogeneous solution, which is then cooled and phase separated. Although many scholars have studied the preparation of UHMWPE microporous membranes by TIPS, the flux of microporous membranes is relatively low because the discontinuous membrane pores are usually closed or semi-closed. This filtration method with a high rejection rate at the expense of flux greatly limits the development of UHMWPE microporous membranes.^23–28^ Therefore, we wanted to design a composite membrane with fibers as the support skeleton and construct a small and dense network structure between the skeletons. On the one hand, the 3D cobweb structure formed by the UHMWPE component in the composite membrane could greatly improve the filtration accuracy. On the other hand, the nonwovens not only act as porous supporting skeletons to increase the strength, but can also penetrate the closed/semi-closed membrane pores of UHMWPE prepared by TIPS, which can effectively improve the water flux. Moreover, the spider web structure prepared by this method is simpler and more convenient than the current mainstream electrospinning spider web structure, and can even realize industrialization.

Herein, we demonstrate a robust strategy to prepare highly effective filtration cotton nonwoven/UHMWPE composite membranes with dual-network structures. The cotton nonwovens were obtained as a fiber skeleton, a 3D arachnoid structure composed of UHMWPE which induced by thermal phase separation was constructed between the skeletons. In addition, due to the hydrophobic surface and low surface energy of UHMWPE, a hyperbranched polymer (HBP) was used to study the hydrophilic modification of the composite membrane surface. Thus, this innovative composite membrane is composed of a 3D nonwoven network and a UHMWPE nano scaffold network. The obtained composite membrane exhibits the integrated properties of small pore size, high porosity and filtration efficiency.

## 2. Experimental

### 2.1. Materials

UHMWPE with *M*_n_ of 4.0 × 10^6^ was provided by Shanghai Lianle Chemical Technology Co., Ltd. Liquid paraffin (LP) was provided by Sinopec Hangzhou Refinery. Dichloromethane (CH_2_Cl_2_) was purchased from Xilong Science Co., Ltd, dopamine hydrochloride was produced by Shanghai Aladdin biochemical Technology Co., Ltd, and tris(hydroxymethyl aminomethane) was provided by National Pharmaceutical Group Chemical Reagent Co., Ltd. In order to evaluate the pore size of membranes, monodisperse polystyrene spheres with particle sizes of 50 nm, 100 nm and 200 nm were prepared by Wuxi Ruige Biotechnology Co., Ltd. Tetraethylpentamine, methyl acrylate, zinc nitrate and dodecanoic acid were provided by Sigma Aldrich. Pure cotton spunlaced nonwovens were purchased from the market.

### 2.2. Preparation of the nonwoven/UHMWPE composite membrane

LP was uniformly blended with a UHMWPE powder in a twin-screw extruder to form a UHMWPE/LP premix with a solid content of 5 wt%. A melted UHMWPE/LP premix was placed in the mold. The premix with a flat vulcanizer was pressed into a film at 200 °C, and then taken out and cooled in water to make a UHMWPE/LP gel film. A 150 × 150 mm pure cotton spunlaced nonwoven net was sandwiched between two layers of gel films and then put on a flat vulcanizer, so that the gel penetrated into the fabric and wrapped the fiber, changing the composite into a film, and then cooled in water to obtain a nonwoven/UHMWPE/LP composite gel film. The composite membrane was immersed in CH_2_Cl_2_ and LP was extracted by ultrasonic extraction for four times, 10 min each time, to obtain the nonwoven/UHMWPE composite membrane, which is stored in absolute ethanol for standby.

### 2.3. Hydrophilic modification of the nonwoven/UHMWPE composite membrane

Tris was used to configure the buffer of pH = 8.5, and dopamine hydrochloride was dissolved in the buffer at a concentration of 1.0 g L^−1^. After dopamine was completely dissolved, the nonwoven/UHMWPE composite membrane was put into it and oscillated at 60 °C for 6 h. After the oscillation, the composite membrane was taken out and washed repeatedly with deionized water to remove the unreacted dopamine on the surface. HBP was prepared according to the method developed by Zhang *et al.*^29^ Then, the HBP with concentrations of 3.0 g L^−1^, 5.0 g L^−1^ and 7.0 g L^−1^ was dissolved in the buffer. After it completely dissolved, it was put it into the composite membrane and oscillated at 60 °C for 24 h.

### 2.4. Characterization of membrane physicochemical properties

The surface morphology and cross-sectional structure of nonwoven/UHMWPE composite membranes were observed using a scanning electron microscope (Gemini SEM30, ZEISS, Germany). Fourier transform infrared (FT-IR) spectra were recorded to characterize the surface chemical properties of composite membranes using an attenuated total reflectance-Fourier transform infrared (ATR-FTIR) spectrometer (i410, thermo, USA). The relationship between mass and temperature was determined using a thermogravimetric analyzer (STA449F5, NETZSCH, Germany) to characterize the stability of the chemical structure. Under the flow of nitrogen, the pure UHMWPE membrane and nonwoven/UHMWPE composite membrane were heated in a crucible at a heating rate of 10 °C min^−1^ and a temperature range of 30 °C–800 °C. A plastic tensile strength instrument (XJ810, Shanghai Xiangjie instrument Technology Co., Ltd) was used to record the tensile measurements at room temperature, a cross-head speed of 100 mm min^−1^, and gauge length and width of 30 and 50 mm, respectively. The contact angle was measured using a contact angle measuring instrument (SDC-350, Dongguan Shengding Precision instrument Co., Ltd) to characterize the hydrophilicity of the modified composite membrane, and the droplet volume was 3 μL. The sample was cut into a round piece with a radius of 10 mm, and 0.5 mL bacterial solution was taken and smeared evenly on the agar. Then, the sample was stuck gently on the agar and cultivated in an incubator at 37 °C for 18–24 hours, and bacterial growth was observed around the sample.

### 2.5. Membrane filtration performance

**Fig. 1:**
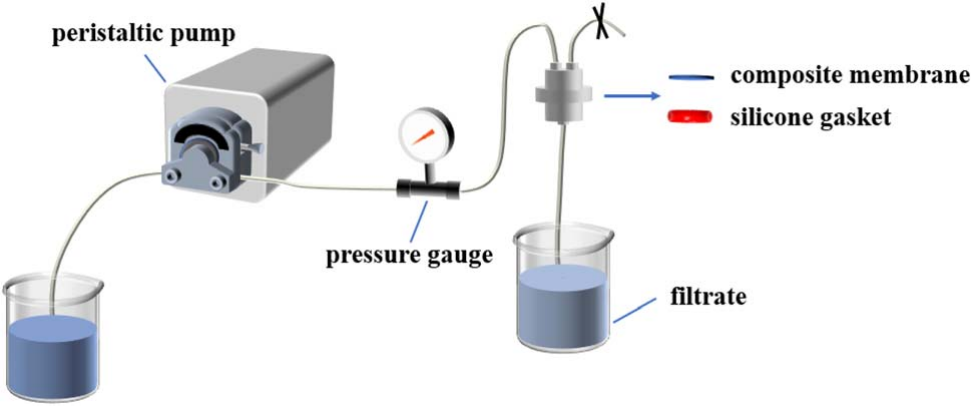
Schematic diagram of the cross flow device.

The filtration performance of composite membranes was tested using a laboratory self-made instrument, including pure water flux and the interception rate of monodisperse polystyrene (PS) nanoparticles. The schematic diagram of the self-made cross-flow filter is shown in Fig. 1. The filtration pressure is 0.1 MPa. After pre-pressing for 30 min, the volume of the filtrate was recorded once every 5 min. The calculation formula of pure water flux *J* (L m^−2^ h^−1^) is given by eqn (1):1
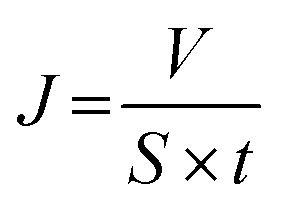
where *V* is the volume of pure water passing through time (L), *S* is the effective filtration area of the composite membrane (m^2^), and *t* is the test time (h).

## 3. Results and discussion

### 3.1. Characterization of the physicochemical properties of composite membranes

**Fig. 2:**
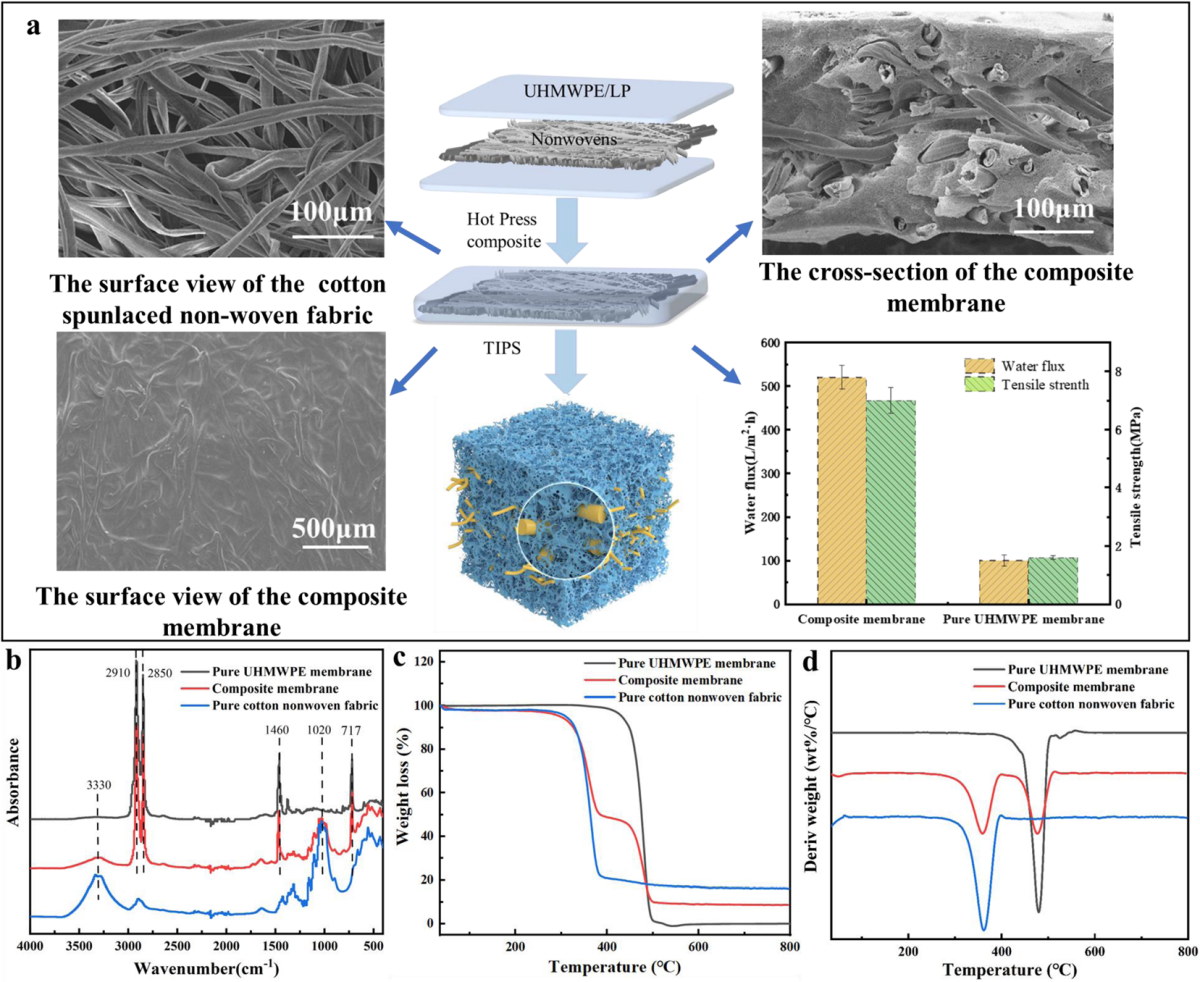
(a) Schematic illustration of the preparation of nonwoven/UHMWPE composite membranes and basic characterization. (b) FTIR-ATR spectra. (c) TG curve. (d) DTG curve.

In this work, a high-performance composite membrane based on dual-network structured nonwoven net/UHMWPE nanopores was constructed *via* a thermal phase separation and composite technique. By thermal phase separation of ultra-high-molecular weight polyethylene (UHMWPE)/liquid paraffin (LP) liquids, this approach enables 3D nanopores to tightly bond with a nonwoven net to form a dual-network structure, which could be clearly seen in Fig. 2(a). Compared with pure cotton spunlaced nonwovens, after combination with the composite, a layer of membrane can be seen on the surface, and the cross-section of the composite membrane showed that 3D nanopores induced by TIPS tightly bonded with the nonwoven net to form a dual-network structure. This unique structure endowed the membrane with excellent filtration properties, compared with the pure UHMWPE membrane, and the pure water flux and breaking strength of the composite membrane increased 5–6 times.

The ATR-FTIR spectrum curve and TG and DTG curves displayed in Fig. 2(b)–(d) also indicate this unique composition. Compared with pure cotton nonwovens, the curve of the composite membrane displayed in Fig. 2(b) shows a typical high and sharp polyethylene absorption peak, and the peaks at 2910 cm^−1^ and 2850 cm^−1^ represent the asymmetric stretching vibration and symmetric stretching vibration of –CH_2_–, 1470 cm^−1^ represents the bending vibration of –CH_2_–, and 717 cm^−1^ represents the in-plane rocking vibration of –CH_2_–. After composition, the broad and strong peaks at 3500–3000 cm^−1^ in the curve of the composite membrane represented the stretching vibration of cellulose –OH of cotton fiber in nonwovens, while 1745 cm^−1^ and 1640 cm^−1^ are the stretching vibrations of lipids and protein –C=O in cotton fibers, respectively. TG and DTG curves displayed in Fig. 2(c) and (d) also indicate the same phenomena. The thermal decomposition process of the composite membrane could be divided into two stages: the initial degradation temperature of the first stage was about 251 °C, which was caused by the thermal decomposition of pure cotton components in the composite membrane, and the initial degradation temperature of the second stage was about 400 °C, which was consistent with the initial temperature of degradation in the curve of the pure UHMWPE membrane. After composition, this dual-network structure endowed the composite membrane with the significantly increased filtration performance; as shown in Fig. 2(a), the water flux and tensile strength of composite membrane had multiplied 5–6 times.

### 3.2. Filtration performance of the composite membrane

In the process of preparing composite membranes, nonwovens and UHMWPE/LP gel films were sandwiched to fabric a composite membrane; after TIPS, nonwovens and UHMWPE act as the net and porous structure of the dual-network respectively. Therefore, the effects of the gram weight of nonwovens and the weight of the UHMWPE/LP gel on the filtration performance of composite membranes were investigated. First, the gram weights of nonwovens of 30 g m^−2^, 40 g m^−2^ and 50 g m^−2^ respectively were selected as a single factor to discuss their effects on the filtration performance of the composite membrane.

**Fig. 3:**
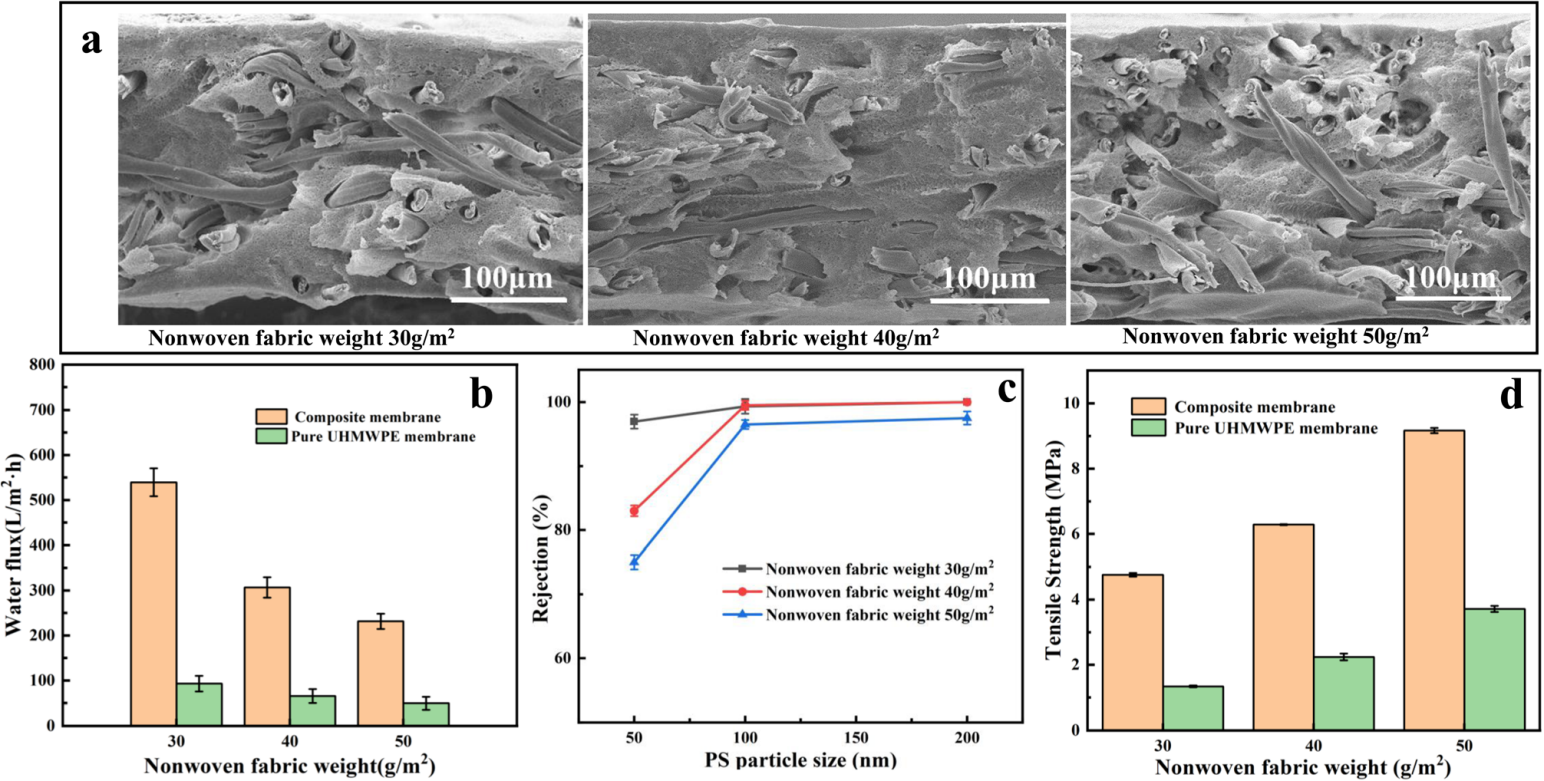
Effects of the gram weight of nonwovens on the composite membrane: (a) SEM images, (b) pure water flux curves, (c) PS nanoparticle rejection rate, and (d) tensile strength.

As shown in Fig. 3(a), three kinds of nonwovens all successfully combined with UHMWPE to construct a three-dimensional penetration porous structure, and with the increase in the weight of nonwovens, the thickness of the composite membrane increased accordingly, which caused a decrease in the membrane water flux (Fig. 3(b)). Compared with the pure UHMWPE membrane of the same thickness, after combination with the composite, the water flux of the composite membrane increased 5–6 times due to the fabrication of this unique dual-network structure. According to eqn (1), the flux is inversely proportional to time. As the membrane thickness increases, the time required for water to flow through complex membrane pores with longer paths increases, leading to a decrease in flux. Hence, with the increase in the gram weight of nonwovens, the thickness of the composite membrane increased accordingly, which caused a decrease in the membrane water flux. However, the increase in thickness did not affect the rejection rate of the composite membrane, as shown in Fig. 3(c). As the fiber skeleton of the main body increases, the pore size of PS ball to pass through the composite membrane increases. As for the water flux, the thickness is the main influencing factor, which can be proved by the fact that the water flux of nonwovens with a higher gram weight is larger than that of UHMWPE/LP with a lower gel weight. Meanwhile, it is found that the breaking strength of the composite membrane increases with the increase in the gram weight of nonwovens, and the breaking strength of the composite membrane also increases significantly compared with the pure UHMWPE membrane, because the stress is mainly borne by the nonwovens as the skeleton during tension, while the fibers in the composite nonwovens are wrapped by UHMWPE, and the stress is transferred from the nonwovens to the UHMWPE. Due to the bonding between UHMWPE and the fibers, the friction between fibers becomes larger and the force required for fracture becomes greater, resulting in a significant increase in the fracture strength after combination with the composite.

**Fig. 4:**
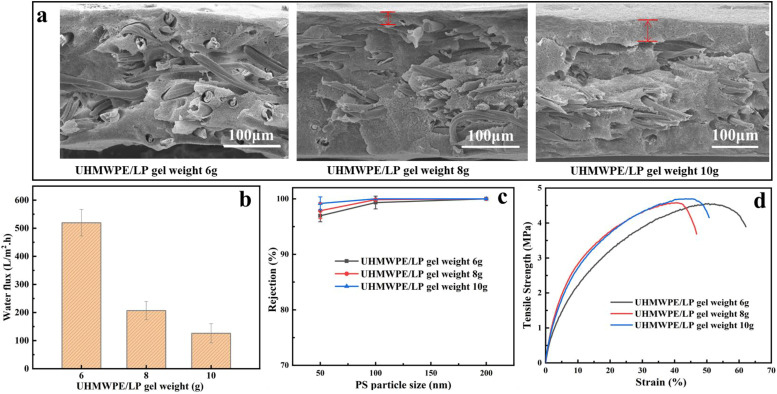
Effects of the UHMWPE/LP gel weight on the composite membrane: (a) SEM images, (b) pure water flux curves, (c) PS nanoparticle rejection rate, and (d) tensile strength.

Next, the effect of the UHMWPE/LP gel weight was studied while the gram weight of nonwovens was 30 g m^−2^. It can be observed from Fig. 4(a) that, after the UHMWPE/LP gel weight increased from 6 g to 8 g and 10 g, a distinct skin was formed with a thickness of 10 to 50 μm, which caused a significant decrease in the water flux of the pure membrane. Meanwhile, it could be seen clearly that the rejection rate was not immune to the mass of the UHMWPE/LP gel. This is because the rejection rate of the membrane is mainly affected by the surface membrane pore size controlled by TIPS, and the increase in UHMWPE/LP gel weight affects only the skin thickness. Therefore, with the increased in UHMWPE/LP gel weight, the rejection rate of the composite membrane does not change much. As shown in Fig. 4(d), the tensile strength was not significantly impacted by the UHMWPE/LP gel weight, because during tension, the stress was mainly borne by the nonwovens as the skeleton, while the fibers in the composite nonwovens are wrapped by UHMWPE, and the stress is transferred from the nonwovens to the UHMWPE, resulting in an inconspicuous change in the tensile strength with the increase in UHMWPE/LP gel weight.

### 3.3. Characterization properties of modified composite membranes

**Fig. 5:**
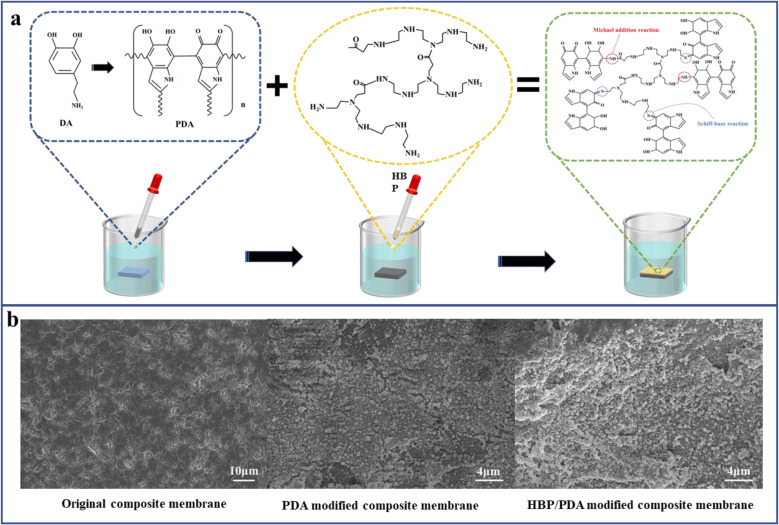
Hydrophilic modification of the nonwoven/UHMWPE composite membrane: (a) schematic diagram and (b) SEM images.

**Fig. 6:**
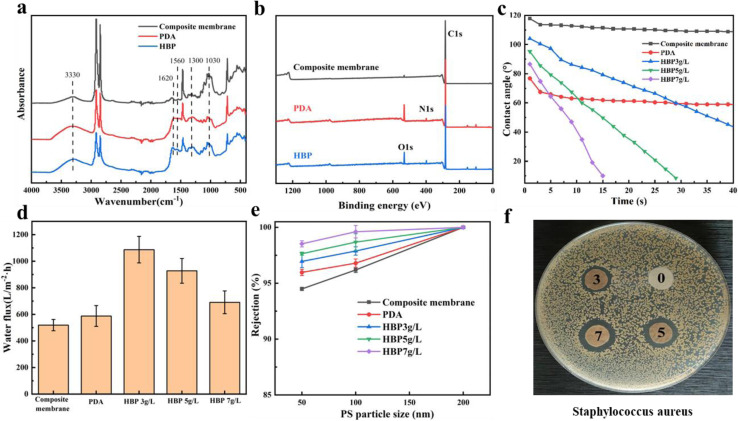
Hydrophilic modification of the nonwoven/UHMWPE composite membrane: (a) FTIR-ATR spectra, (b) XPS spectra, (c) dynamic contact angle, (d) pure water flux curves, (e) PS nanoparticle rejection rate, and (f) bacteriostatic circle of *S. aureus*.

The previous section showed the successful fabrication of a dual-network structure, which endowed the composite membrane with an outstanding pure water flux. However, the composition did not change the hydrophobicity of the nonwoven/UHMWPE composite membrane, and UHMWPE exhibits strong hydrophobicity due to the linear nonpolar chain structure; numerous researchers^30–36^ have proved that improving the hydrophilicity of the membrane surface could effectively improve the membrane filtration performance. Hence, a layer-by-layer self-assembly hydrophilic surface was constructed on the membrane surface to further enhance the membrane filtration performance. The mechanisms of a layer-by-layer self-assembly hydrophilic surface are shown in Fig. 5(a). First of all, dopamine self-polymerization and firm attachment to composite membrane surfaces, and subsequently, the HBP covalently bind with the reactive quinone structure on the PDA layer *via* a Schiff base or a Michael addition base. The SEM images of the composite membrane surface modified by dopamine and HBP are shown in Fig. 5(b); compared with the porous structure on the original composite membrane, after dopamine self-polymerization on the membrane surface, an typical nanosphere PDA layer was observed on the membrane surface, and after a layer-by-layer stacked HBP was formed, the layer on the membrane surface became thicker, the nanospheres on the membrane surface became larger and denser, and the surface pores of the membrane were distinctly diminished, as the repulsion between the terminal hydrophilic cationic groups on the HBP chain spontaneously stretches to form a spherical structure.^37^ The coverage of the HBP/PDA layer on the composite membrane surface reduces the surface pore size and increases the membrane hydrophilic, which will affect the membrane water flux and BSA rejection rate. Fig. 6(a) shows the FTIR spectra of the composite membrane surface after coating with PDA and immobilizing with the HBP. After dopamine modification, a higher and wider–NH–/–OH stretching vibration peak appears at 3500–3000 cm^−1^, which is caused by aryl secondary amine and heterocyclic secondary amine in dopamine. The shoulder peaks at 1610 cm^−1^ and 1519 cm^−1^ indicate the overlapping vibration of the –C–C– bond and the shear vibration of–NH–, respectively. The stretching vibration of –CO– is at 1300 cm^−1^, and the peaks near 1030 cm^−1^ are assigned to –OH–, –CH– and –COC– groups in dopamine. However, the ATR curve of HBP is mainly an enhancement peak on the basis of dopamine curve at 1750–500 cm^−1^, for which the in-plane vibration peak of secondary amines unique to HBP appears at 1617 cm^−1^ and 1560 cm^−1^. Fig. 6(b) shows the wide scans for the composite membrane surface after coating with PDA and immobilizing with the HBP. Compared with the original composite membrane, new O 1s and N 1s peaks appeared on the spectrum of PDA and PDA/HBP modified membranes, which are attributed to oxygen and nitrogen from the PDA layer and HBP layer, indicating the successful layer-by-layer self-polymerization on the composite membrane surface.

The dynamic water contact angle measurements were employed here to characterize the hydrophilicity and wetting ability of the PDA and PDA/HBP modified membranes. As shown in Fig. 6(c), the water contact angle of the original non-woven/UHMWPE composite membrane was as high as 117.8°, and after modification with dopamine, the contact angle decreased to 67.8°. The dynamic change in the contact angle was remarkable after layer-by-layer stacking of the HBP, and within one minute, the contact angle gradually decreased to 0, showing excellent hydrophilicity and wettability. This is due to the amino, hydroxyl, amide bond and other polar groups on the HBP layer, which improve the surface wettability and decrease the free energy of the composite membrane surface. From the above-mentioned characterization results, it could be seen that after the self-assembly of dopamine and HBPs on the membrane surface, the surface pore size decreased, and the surface hydrophilicity and wettability increased, which will bring a new leap for the filtration performance of the composite membrane. As shown in Fig. 6(d) and (e), the PDA/HBP-modified membrane performed particularly well in the pure water flux and PS nanoparticle rejection rate. Research shows that the pure water flux was mainly affected by the membrane surface hydrophilicity and wettability. For the HBP-immobilized composite membrane, the composite membrane surface hydrophilicity was remarkably increased, the pure water flux increased from 519 L m^−2^ h^−1^ to 1054 L m^−2^ h^−1^, and the water flux decreased with the increased HBP content, as the excessive polymerization of HBP on the membrane surface blocked the pore structure on the membrane surface and led to a decrease in the pure water flux. Hence, the optimal HBP immobilizing content was 3% for the composite membrane. It is also clear that the rejection rate of PS nanospheres with different particle sizes increased after HBP immobilization, and it increased with the increase in HBP content. This higher PS nanosphere rejection rate was due to the decreased pore size on the membrane surface, as observed in Fig. 5(b). Furthermore, the HBP modified composite membrane also showed excellent antibacterial performance against *Staphylococcus aureus* (*S. aureus*), as observed in Fig. 6(f). It could be observed from Fig. 6(f) that there was no bactericidal activity towards *S. aureus* for the virgin composite membrane (0). However, the HBP/PDA-modified composite membrane (3, 5, and 7) had shown antibacterial properties under the same condition, because the terminal amino hyperbranched polymer has antibacterial properties. Numerous reports^37–39^ have proved that the physical barrier and electrostatic repulsive force of hydrated layer formed by the positively charged hyperbranched terminal amino polymer hydrophilic surface can block bacteria while destroying the negatively charged bacterial cell membrane, thus showing good antibacterial performance. In the water treatment process, the accumulation and growth of bacteria on the membrane surface will block the membrane pores, resulting in a decrease in filtration efficiency. Hence, the layer-by-layer self-polymerization of PDA/HBP on the membrane surface not only effectively increased the membrane flux and improved the rejection rate, but also endowed the composite membrane with antibacterial functions.

## 4. Conclusion

In this work, a high-throughput and efficient filtration non-woven/UHMWPE composite membrane has been prepared by hot pressing and TIPS. Next, a layer-by-layer self-assembly hydrophilic surface has been constructed on the membrane surface to further enhance the membrane filtration performance. The effects of UHMWPE/LP gel weight, gram weight of nonwovens and HBP concentration on the filtration performance of the composite membrane were discussed. The result indicated that the dual-network structured could effectively improve the pure water flux of the composite membrane, compared to the pure UHMWPE membrane; the pure water flux of the composite membrane increased 6–8 times, however, compared to the pure cotton spunlaced fabric, the filtration precision of the membrane changes from microfiltration to nanofiltration after composition. Moreover, the layer-by-layer self-polymerization of PDA/HBP on the membrane surface not only effectively increased the membrane flux and improved the rejection rate, but also endowed the composite membrane with antibacterial functions; the pure water flux increased from 519 L m^−2^ h^−1^ to 1054 L m^−2^ h^−1^ and the rejection rate of 50 nm PS nanospheres was almost close to 100%. This simple and effective technology significantly broadens the application prospects of nonwoven filter materials and provides new insights into the design and development of high-performance filtration and separation materials for various applications.

## Conflicts of interest

There are no conflicts to declare.

## Supplementary Material
